# The amino acid and carnitine concentration changes in bronchoalveolar lavage fluid from lung cancer patients

**DOI:** 10.1186/s12957-022-02850-5

**Published:** 2022-12-04

**Authors:** Xiaojun Sun, Mengmin Xu, Liren Ding, Guobiao Yang, Jianlong Kong, Yafang Zhu, Xuefang Chen, Liang Xu, Yisha Shan, Yupin Xu

**Affiliations:** 1 Department of Respiratory Medicine, Affiliated Hospital of Shaoxing University, Shaoxing, 312000 Zhejiang Province China; 2 School of Medicine, Shaoxing University, Shaoxing, 312000 Zhejiang Province China; 3 Department of Respiratory Medicine, The Second Affiliated Hospital of Zhejiang University School of Medicine, Hangzhou, 311009 Zhejiang Province China; 4 Zhejiang Huayang Pharmaceutical Co., Ltd., Hangzhou, 311009 Zhejiang Province China; 5 Hangzhou Beijian Biotechnology Co., Ltd, Hangzhou, 311009 Zhejiang Province China

**Keywords:** Lung cancer, BALF, Targeted metabolomics, Biomarker

## Abstract

**Objective:**

To screen out potential biomarkers by analyzing fundamental nutrients in the bronchoalveolar lavage fluid (BALF) before confirming the lung cancer.

**Methods:**

In this study, 44 patients were enrolled with clinical information. The concentrations of 23 amino acids and 35 carnitines in their BALF were detected with the high-performance liquid chromatography–mass spectrometry (HPLC–MS). Combined with clinicopathological diagnosis, the patients were divided into the lung cancer group (grades I & II and III & IV) and the non-cancer group for standard statistical analysis.

**Results:**

The partial least squares-discriminant analysis (PLS-DA), the Shapiro–Wilk test, and the Bonferroni correction results showed that the serine concentration was higher and the butane-diacyl-carnitine (C4DC) concentration was lower in the lung cancer group, further showing the same changing trend continuously through the non-cancer stage, grades I & II stage and grades III & IV stage. Those two potential biomarkers have been identified.

**Conclusion:**

The HPLC–MS target detection in clinic for nutrient concentration levels is a promising technique to find the changing concentration of serine and C4DC in BALF, which provides an economical and practical way for early warning of lung cancer.

**Supplementary Information:**

The online version contains supplementary material available at 10.1186/s12957-022-02850-5.

## Introduction

According to a recent report on cancer statistics, primary lung cancer is a worldwide disease, ranking the top in mortality and cancer-related deaths in men and women [1]. In a recent statistical study, the incidence of lung cancer is 0.45‰ for Chinese men, 0.27‰ for Chinese women in 2015, with the highest mortality (0.38‰ for men and 0.14‰ for women) among various cancers [2]. Most patients with the early lung cancer have no specific clinical symptoms, and their clinical confirm is too late for their treatment. The 5-year survival rate of lung cancer in China without surgical treatment is only 16.1%, which is far lower than that in developed countries [3].

Early tests and diagnosis are the key to reduce the mortality of lung cancer, and early lung cancer patients with clinical treatment can obtain good prognosis. However, lacking of clinical symptoms at an early stage, most patients (75%) are generally confirmed advanced lung cancer (III/IV period) and miss the best treatment [4]. In 2011, the National Lung Screening Trial reported that low-dose computed tomography (LDCT) examination significantly reduces lung cancer mortality in high-risk populations [5]. In clinical CT and LDCT examination, the result of false positive is still a problem unsolved, and the early screening and diagnosis only relying on pulmonary imaging is insufficient [6, 7]. With the development of molecular biology, tumor biomarkers are of great clinical value for screening malignant tumors, such as cancer embryonic antigen (CEA), neuron-specific enolase (NSE), cytokeratin fragment antigen21-1 (CYFRA21-1), and squamous cell carcinoma-related antigens, which are widely used in clinical practice [8].

Metabolomics, an important means of translational medical research, is used to study the pathogenesis and biomarkers of lung cancer [9]. The metabolomics result can reflect metabolite changes in specific pathophysiological conditions, providing a new research way for disease diagnosis [10]. At various stages of malignant tumors, metabolomic provides the potential mechanism by the metabolic characteristics of tumors [11]. The complex metabolic network consisting of proinflammatory cytokines, neuroendocrine hormones, neurotransmitters, eicosanoids, and tumor-derived factors produced by the body in response to the tumor and by the tumor itself, which contributes to the humoral changes in cancer microenvironment [12]. Among metabolites, amino acids and carnitines are some of the most suitable biomarkers for researching cancer because they are ingested and synthesized directly in fundamental metabolism, as basic nutrients supplying tumor cells.

In this study, we collected the BALF from patients with tracheal examination before their lung cancer was confirmed and analyzed the metabolites (including 23 amino acids and 35 carnitines) in BALF by HPLC–MS, got 35 values of metabolite ratio, built the PLS-DA model and found potential biomarkers. It is potential to find biomarkers correlated to lung cancer in routine clinical tests of the BALF as an experimental basis for early cancer warning.

## Materials and methods

### Study population and sample collection

During the period from April 2017 to March 2020, 44 patients were enrolled in this study and their clinical data were collected from the medical records in Affiliated Hospital of Shaoxing University, Shaoxing, Zhejiang Province, China. All lung cancer patients were diagnosed by pathological and cytological tests. Patients with severe metabolic diseases or other cancers were excluded in the study. Tracheal examination was performed in accordance with routine preoperative requirements and BALF samples were collected in clinical examinations. Sample transfer, centrifugation, and separation were completed within 3 h to avoid any preanalytical factors that might affect amino acid and carnitine concentrations. Samples were stored at − 80 °C until analysis.

### Amino acid and carnitine measurement

#### Chemicals

The succinylacetone, non-derived amino acid, and carnitine assay kit (NZP108) was purchased from Guangzhou Fenghua Bioengineering Co., Ltd. (Guangzhou, China). This assay kit was mainly used for diagnosing metabolic diseases in clinic and the PR China Medical Device Registration Certificate Number was 2016-3-40-1324.

The detected amino acids included alanine (Ala), arginine (Arg), asparagine (Asn), aspartic acid (Asp), citrulline (Cit), cysteine (Cys), glutamic acid(Glu), glutamine (Gln), glycine (Gly), homocysteine (Hcy), histidine (His), leucine (Leu), lysine (Lys), methionine (Met), ornithine (Orn), phenylalanine (Phe), piperine (Pip), proline (Pro), serine (Ser), threonine (Thr), tryptophan (Trp), tyrosine (Tyr), and valine (Val); the detected carnitines included free carnitine (C0), ethane-acyl-carnitine (C2), propane-acyl-carnitine (C3), propane-diacyl-carnitine (C3DC), butane-acyl-carnitine (C4), trihydroxy-butane-acyl-carnitine (C4OH), butane-diacyl-carnitine (C4DC), isopentane-acyl-carnitine (C5), trihydroxy-isopentane-acyl-carnitine (C5OH), pentane-diacyl-carnitine (C5DC), isopentene-acyl-carnitine (C5:1), hexane-acyl-carnitine (C6), hexane-diacyl-carnitine (C6DC), octane-acyl-carnitine (C8), decane-acyl-carnitine (C10), decene-acyl-carnitine (C10:1), decadiene-acyl-carnitine (C10:2), dodecane-acyl-carnitine (C12), tetradecane-acyl-carnitine (C14), trihydroxy-tetradecane-acyl-carnitine (C14OH), tetradecane-diacyl-carnitine(C14DC), tetradecene-acyl-carnitine (C14:1), tetradecadiene-acyl-carnitine (C14:2), hexadecane-acyl-carnitine (C16), trihydroxy-hexadecane-acyl-carnitine (C16OH), trihydroxy-hexadecene-acyl-carnitine (C16:1OH), octadecane-acyl-carnitine (C18), trihydroxy-octadecane-acyl-carnitine (C18OH), octadecene-acyl-carnitine (C18:1), trihydroxy-octadecene-acyl-carnitine (C18:1OH), octadecadiene-acyl-carnitine (C18:2), eicosane-acyl-carnitine (C20), doeicosane-acyl-carnitine (C22), tetraeicosane-acyl-carnitine (C24), and hexaeicosane-acyl-carnitine (C26); the metabolite ratios included Arg/Orn, Cit/Arg, Gly/Ala, Met/Leu, Met/Phe, Orn/Cit, Phe/Tyr, Tyr/Cit, Val/Phe, C2/C0, C3/C0, C3/C2, C3/C16, C4/C2, C4/C3, C4/C8, C5/C0, C5/C2, C5/C3, C5-OH/C8, C5-OH/C0, C5DC/C5-OH, C5DC/C16, C8/C2, C8/C10, C16-OH/C16, C26/C20, C14:1/C16, C3DC/C10, C10:2/C10, C5DC/C8, (C0 + C2 + C3 + C16 + C18:1)/Cit, (C16 + C18)/C0, C0/(C16 + C18), and C3/Met; HPLC-grade methanol was purchased from Merck (Darmstadt, Germany); AR-grade trichloromethane was purchased from Shanghai Lingfeng Chemical Reagent Co., Ltd. (Shanghai, China).

#### Sample preparation

One hundred microliters of BALF sample was added to 400 μL deproteinizing solution (methanol: trichloromethane, 9:1) with a mixed internal standard (amino acid and carnitine isotopic internal standard products from the assay kit). The mixture was vortexed for 1 min and then centrifuged at 13,000 rpm at 4 °C for 10 min. The 300 μl supernatant of mixture was lyophilized and then it was redissolved in 100ul extraction liquid (containing methanol and water from the assay kit). It was completely dissolved by ultrasonic at 45 ℃ for 45 min and 10 μL was used for LC–MS/MS analysis.

#### LC–MS/MS analysis

Amino acid and carnitine concentrations were performed by the Waters Acquity UPLC I-Class/Xevo TQD system (Waters, USA) and the LC–MS/MS detecting procedure followed the assay kit instruction. The assay kit offered the customized mobile phase and flow rate was 0.2 mL/min. The multiple reaction monitoring (MRM) and neutral loss scanning pattern were performed by mass spectrometry without UPLC separation, referring to the assay kit condition.

#### Quality control (QC)

During detecting process, there were 2 blank samples, 2 low concentration QC samples, and 2 high concentration QC samples in the 96-well plate, and the rest were the BALF samples. Because the platform was also used for a large amount of clinical blood samples with the same assay kit, all quality control sample data on that day were listed (Table S1).

According to the QC requirement from this assay kit, the recovery of various amino acids and carnitines with isotopic internal standard products must be 80–120%, and variable coefficient of QC sample concentration must be less than 20%. When the concentration value of high or low concentration QC samples conforms to the standard value ± 3 standard deviation, the concentration values of BALF sample are considered to be effective; otherwise, they need to be detected again.

### Data analysis

Using SPSS20.0 (IBM, USA) in the study was for basic data analysis. The Shapiro–Wilk test was used to test for normality, then the Mann–Whitney *U* test was performed for between group comparisons, in cases of two groups of continuous variables. And the Bonferroni correction was used to counteract the problem of multiple comparisons. The curves of receiver operating characteristic (ROC) were obtained the clinical diagnosis value of metabolites. A multivariate data analysis of PLS-DA was performed using SIMCA-P13.0 (Umetrics, Sweden).

## Results

In the study, 44 patients were enrolled and their BALF samples were collected. Based on their diagnosis, they were divided into two large groups (the lung cancer group and the non-cancer group) then into four specific groups especially. The samples of squamous cell carcinoma and adenomatous carcinoma were set into the cancer group, and the samples of tuberculosis and pneumonia were set into the control group. We collected clinical information on BALF providers, including age, gender, family history, smoking history, and drinking history (Table 1). Patients with lung cancer are generally older than those with pneumonia and tuberculosis and are more likely to be male. Lifestyle habits such as smoking and drinking are strongly linked to lung cancer. Subsequently, we also collected oncology characteristic data (Table 2). Whether malignancy has a strong significance to distinguish the lung cancer grade. The cancer patients with only one primary in IV grade are main individuals in groups.

**Table 1:** Demographic characteristics of patients

Group	Lung cancer (*n* = 22)			Non-cancer (*n* = 22)		
	Squamous cell carcinoma (*n* = 12)	Adenomatous carcinoma (*n* = 10)	Total	Tuberculosis (*n* = 12)	Pneumonia (*n* = 10)	Total
Age (year)	73 ± 6	67 ± 9	68 ± 8	51 ± 14	61 ± 4	57 ± 11
Gender (F/M)	1/11	2/8	3/19	7/5	6/4	13/9
Family history	0	0	0	0	0	0
Smoking history	9	6	15	2	3	5
Drinking history	7	4	11	2	2	4

Data are presented as mean ± standard deviation, *F/M* means males or females

**Table 2:** Oncology characteristics of patients

Group	Squamous cell carcinoma (*n* = 12)	Adenomatous carcinoma (*n* = 10)	Total
Grade			
I	1	1	2
II	2	2	4
III	1	1	2
IV	8	6	14
Primary type			
One primary only	12	9	21
More primaries	0	1	1
In situ/malignant tumor			
In situ	3	3	6
Malignant	9	7	16

**Fig. 1:**
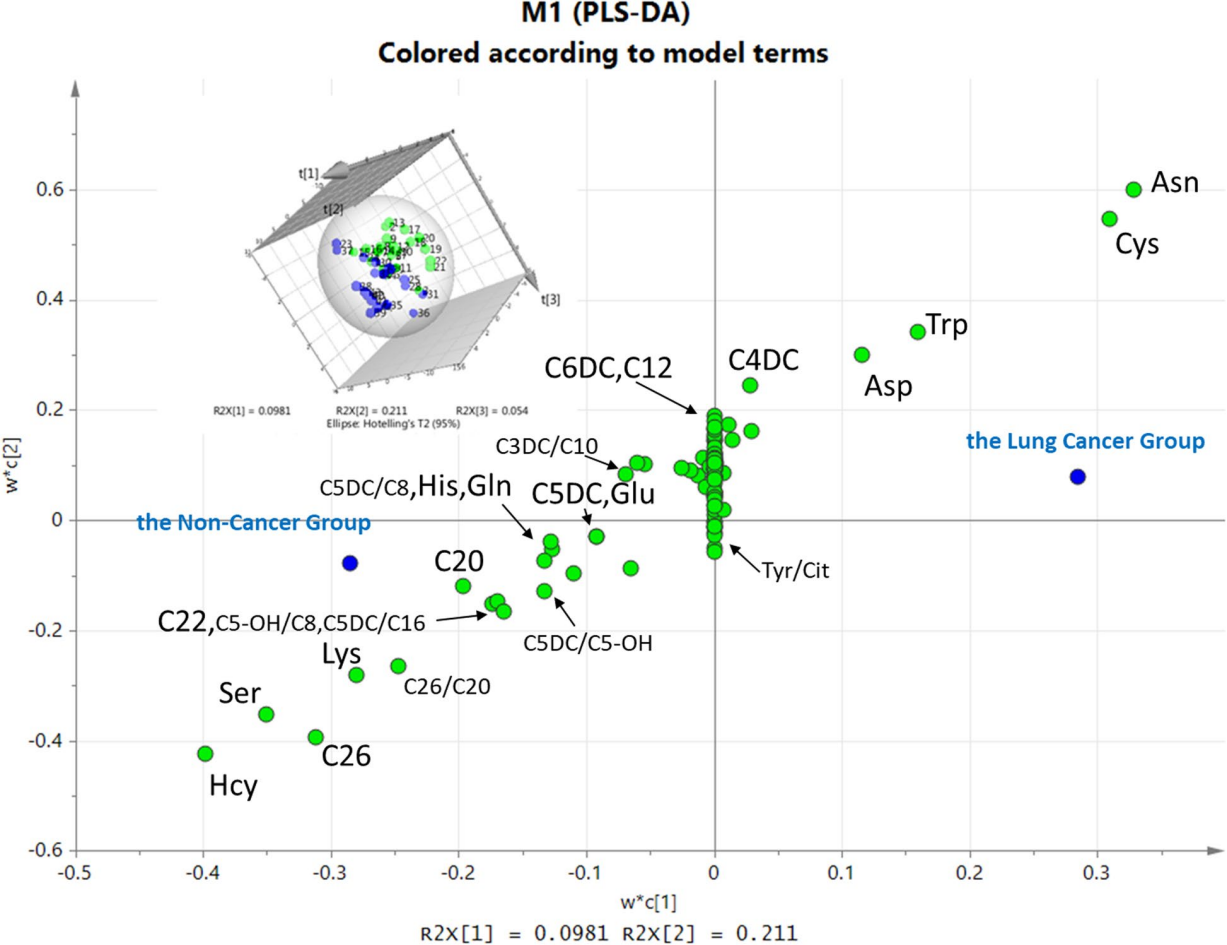
The PLS-DA model of the lung cancer group and the non-cancer group. The small image in the upper left corner is the score plot of the PLS-DA model. The axis represents the principal component. The green scatter represents the non-cancer samples and the blue scatter represents the lung cancer samples. The main image is the loading plot of the PLS-DA model. The axis represents the principal component. The green scatter represents the metabolite and the blue scatter represents the non-cancer group or the lung cancer group. R2Y(cum) is 0.527 and Q2(cum) is − 0.326 in this model

The PLS-DA model was performed on the metabolite concentration and ratio values of the two groups. In the score plot of the PLS-DA model (an image in the upper left corner of Fig. 1), sample scatters show that green scatters (the non-cancer samples) and blue scatters (the cancer samples) were effectively distinguished in the 3D rectangular coordinate system, indicating that the metabolite concentration and ratio values in the statistical modeling has discriminant power. The loading plot shows that the included values (green scatters) were distributed between the two groups (two blue scatters).

The metabolites (VIP value > 1) were tested by the Shapiro–Wilk test (Table 3). The concentration of Ser, C26, the metabolic ratio value of C26/C20, C5DC/C5-OH in the lung cancer group were higher than those in the non-cancer group, and the concentrations of Hcy, Asn, Cys, Trp, Glu, Asp, Gln, and C4DC in the lung cancer group were lower than those in the non-cancer group (*p* < 0.01). The metabolite ratio of C5DC/C8, C5-OH/C8 in the lung cancer group were higher than those in the non-cancer group, and the concentrations of Lys, C12 in the lung cancer group were lower than those in the non-cancer group (*p* < 0.05).

**Table 3:** The list of potential biomarkers from the PLS-DA model

Metabolite	VIP value	Lung cancer		Non-cancer		*p* value*
		Mean ± SD	*p* value	Mean ± SD	*p* value	
Hcy	2.94	8.45 ± 0.38	< 0.001	8.81 ± 0.39	0.005	0.001
Ser	2.59	3.29 ± 1.16	< 0.001	2.40 ± 1.05	0.002	< 0.001
Asn	2.59	28.84 ± 5.39	< 0.001	37.18 ± 4.09	0.059	< 0.001
Cys	2.41	1.26 ± 0.33	< 0.001	2.77 ± 3.43	< 0.001	0.001
C26	2.37	0.01 ± < 0.01	< 0.001	0.01 ± 0.01	< 0.001	0.001
Lys	2.16	10.52 ± 1.92	< 0.001	42.93 ± 78.79	< 0.001	0.048
C26/C20	1.82	0.25 ± 0.16	< 0.001	0.11 ± 0.21	< 0.001	0.001
C20	1.58	0.02 ± 0.01	< 0.001	0.02 ± 0.02	0.001	0.937
C6DC	1.47	0.06 ± 0.14	< 0.001	0.09 ± 0.17	< 0.001	0.864
Trp	1.39	5.21 ± 1.07	< 0.001	6.80 ± 2.38	0.003	0.001
C5DC/C8	1.35	0.56 ± 0.62	< 0.001	0.54 ± 0.83	< 0.001	0.043
C5-OH/C8	1.33	0.84 ± 0.64	< 0.001	0.57 ± 0.56	0.001	0.025
C4DC	1.31	0.03 ± 0.02	< 0.001	0.05 ± 0.02	0.003	0.001
C5DC/C16	1.31	0.26 ± 0.23	0.001	0.46 ± 0.83	< 0.001	0.590
C22	1.30	0.01 ± 0.01	< 0.001	0.02 ± 0.03	< 0.001	0.433
Glu	1.24	8.43 ± 1.45	< 0.001	11.15 ± 3.91	0.045	0.001
Asp	1.12	6.79 ± 1.81	< 0.001	8.81 ± 3.51	0.009	0.002
Gln	1.11	1.07 ± 0.26	< 0.001	2.68 ± 3.57	< 0.001	0.002
C5DC/C5-OH	1.10	0.91 ± 1.14	< 0.001	0.17 ± 0.32	< 0.001	0.002
C5DC	1.06	< 0.01 ± < 0.01	< 0.001	0.02 ± < 0.01	< 0.001	0.127
C12	1.05	0.01 ± 0.02	< 0.001	0.03 ± 0.04	< 0.001	0.022
C3DC/C10	1.05	0.37 ± 0.29	< 0.001	0.33 ± 0.27	0.005	0.149
His	1.05	7.84 ± 6.50	< 0.001	9.35 ± 13.69	< 0.001	0.360
Tyr/Cit	1.01	3.89 ± 8.26	< 0.001	2.38 ± 2.55	0.001	0.851

*VIP value* variable importance in the projection is assessed using the PLS-DA model, The data are presented as mean ± standard deviation: amine acid and carnitine unit was μmol/L; *p value* the statistical significance was assessed using the Shapiro–Wilk test. p value* the statistical significance was assessed using the Mann–Whitney *U* test. p < 0.05 indicates significance and *p* < 0.01 indicates more significance for statistical analysis. The C26 level of non-cancer group is 0.007 ± 0.010 μmol/L, its lung cancer group 0.010 ± 0.004 μmol/L

Considering the false positive results caused by multiple hypothesis tests, the Bonferroni Correction was used in added statistical analysis. There were 93 comparisons between the two groups in the study, so the cut-off value for statistical significance was < 0.000538, and cut-off value for more statistical significance was < 0.000108. In Table 3, Ser (*p = 0.000063) and Asn (*p = 0.000005) had profound statistical significance.

**Fig. 2:**
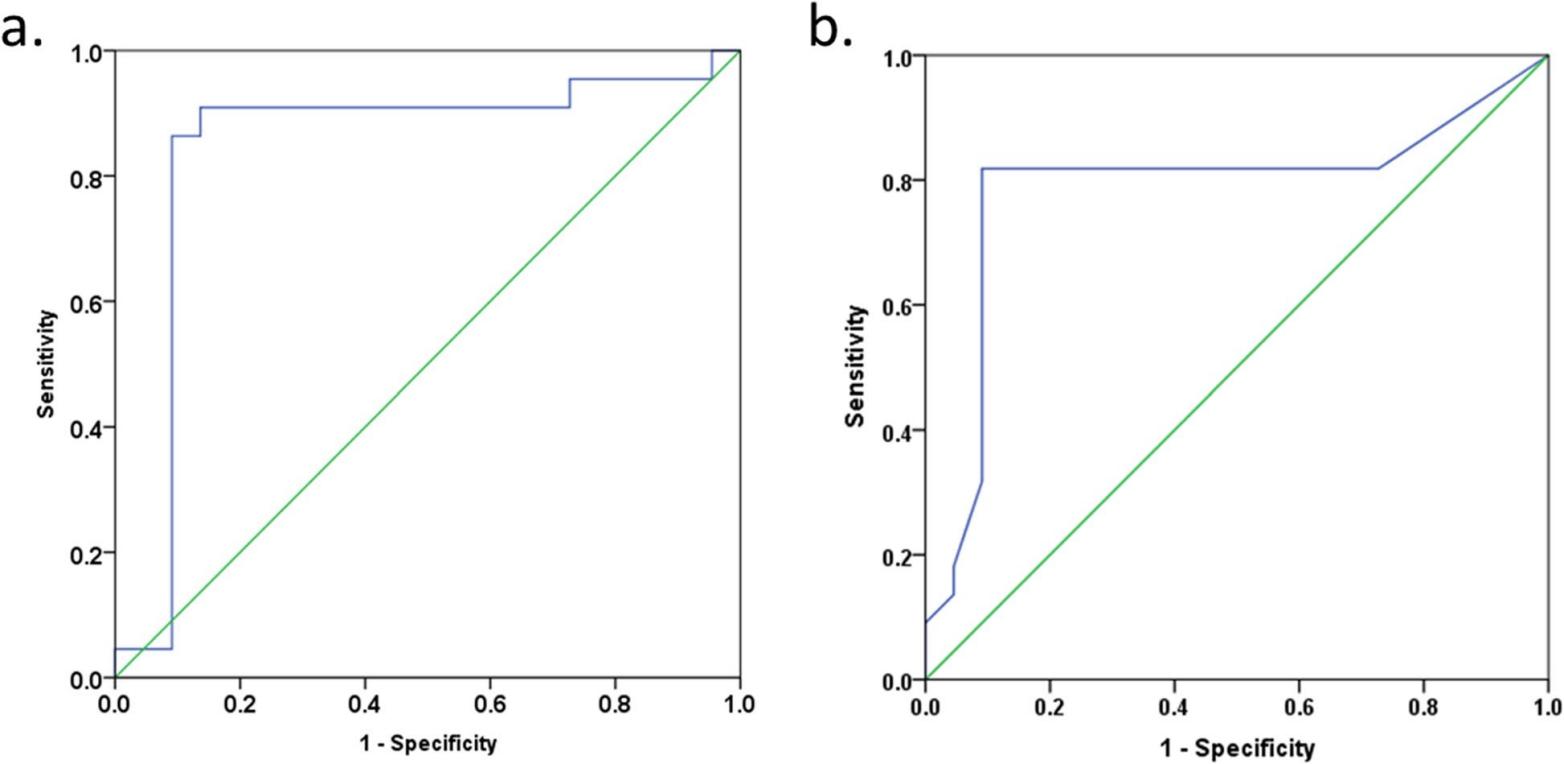
The ROC curve analysis for the discrimination of the lung cancer group from the non-cancer group. **a** The serine concentration. **b** The C4DC concentration

To further explore the relationship between metabolite changing and developed grade, metabolites with more statistical significance had been researched among the non-cancer stage, grades I & II stage and grades III & IV stage (Table 4). The Ser concentration in BALF had higher level as lung cancer progresses, but C4DC was opposite. The area under the curve (AUC) of Ser was 0.843 and that of C4DC was 0.785 (Fig. 2). They differed significantly between the non-cancer group and the cancer group, and the same trend continuously between grades I & II stage and grades III & IV stage. Asp, Glu, and C26 did not have a strong trend continuously between grades I & II stage and grades III & IV stage, and C26/C20 had a strong correlation with C26.

**Table 4:** The list of the more significant PLS-DA model biomarkers linked to cancer grades

Metabolite	Non-cancer (*n* = 22)	Lung cancer		
		Grades I & II (*n* = 6)	Grades III & IV (*n* = 16)	*p* value
	Mean ± SD	Mean ± SD	Mean ± SD	
Asp	8.81 ± 3.51	6.58 ± 2.00	6.87 ± 1.79	0.033
Glu	11.15 ± 3.91	8.20 ± 1.95	8.51 ± 1.28	0.027
Ser	2.40 ± 1.05	3.14 ± 0.22	3.34 ± 1.36	0.017
C4DC	0.05 ± 0.02	0.02 ± 0.02	0.01 ± 0.02	0.013
C26	0.01 ± 0.01	0.01 ± < 0.01	0.01 ± < 0.01	< 0.001
C26/C20	0.11 ± 0.21	0.31 ± 0.10	0.23 ± 0.18	0.003

The data are presented as mean ± standard deviation: amine acid and carnitine unit was μmol/L; *p value* the statistical significance between the grades I & II group and the grades III & IV group was assessed using the Mann–Whitney *U* test. *p* < 0.05 indicates significance and *p* < 0.01 indicates more significance for statistical analysis. Due to reserving two decimal places, the data related to C26 in this table are not clear. The C26 level of non-cancer group is 0.007 ± 0.010 μmol/L, its grades I & II 0.012 ± 0.004 μmol/L and grades III & IV 0.009 ± 0.004 μmol/L

## Discussion

The proliferation of cancer cells needs to consume nutriment and energy from the body, which causes changes in many metabolic pathways [13]. Cancer cells metabolize differently from normal cells, which suggests that specific metabolites may serve as cancer biomarkers [14]. Amino acids and carnitines, as important nutriment in human metabolic pathways, have a potential to be biomarkers for early diagnosis of lung cancer [15, 16]. In this study, we quantified most of the amino acids and carnitines in BALF involved in the body metabolism. Serine and butane-diacyl-carnitine (C4DC) have been found to have potential value as biomarkers through targeted metabonomics.

We found the serine concentration is higher in BALF from lung cancer patients. In physiological conditions, 3-phosphoglycerate dehydrogenase (PHGDH), phosphoserine aminotransferase1 (PSAT1), and phosphoserine phosphatase (PSPH) regulate the production of Ser. However, changes of the de novo serine synthesis pathway in cancer cells are common pathological phenomena [17]. Elevated enzyme expression in the de novo synthesis pathway helps cancer cells survive in serine-deficient microenvironments. Serine is used in the synthesis of membrane lipid component and essential amino acids in this microenvironment [18]. PHGDH and PSAT1 are activated in non-small cell lung cancer, leading to changes in the metabolic pathways of serine [19].

In physiological conditions, asparagine synthetase (ASNS) regulates aspartic acid and glutamine to produce asparagine. But cancer cells lack the ASNS expression and rely on asparagine in the microenvironment [20, 21]. This non-essential amino acid is involved in tricarboxylic acid cycle, such as glutamic acid. It decreases in BALF from lung cancer patients, suggesting changes in energy metabolism.

Carnitine concentrations and their ratio values were different in the BALF between the lung cancer patients and the non-cancer patients in our study, suggesting changes happened possibly in metabolic network. The changes of carnitine metabolism in cancer patients have the potential to be tumor markers [22]. It was worth mentioning that C4DC went down as the cancer progresses (Table 4). Some studies have shown that C4DC came from methylmalonyl-CoA when propionyl-CoA carboxylase worked as a regulator [23]. However, there were also scanty studies on C4DC metabolism.

Compared to previous studies [24, 25], we systematically detected the concentrations of amino acid and carnitine concentrations in the BALF. Asparagine concentration is lower in BALF from lung cancer patients, which has been proved by Callejón-Leblic. In terms of clinical biomarkers, the combined diagnosis of multiple markers has become more important, meaning that it is very urgent to research metabolites by their classification. But the limitations of many factors, the biological mechanism needs to be explored in the future.

## Supplementary Information

**Additional file 1.**

## Data Availability

All the data and material can be available. The data used to support the findings of this study are available from the corresponding author upon request.
