# Supplementary material for: The amino acid and carnitine concentration changes in bronchoalveolar lavage fluid from lung cancer patients
Source: World J Surg Oncol. 2022 Dec 4;20:380. doi: 10.1186/s12957-022-02850-5 (PMC9720910; doi:10.1186/s12957-022-02850-5)
Supplement: Supplementary file 1 — Additional file 1. [file 12957_2022_2850_MOESM1_ESM.docx]

**Table. S1 The List of 10 Amine Acids and 13 Carnitines (μmol/L) in 20 Low Concentration QC Samples and 20 High Concentration QC Samples**

| **Quality Indicator** | | **Test Linear Range (Waters Xevo TQD)** | **Target Value** | **The Test Values of Quality Control Sample** | | | | | | | | | | | | | | | | | | | | **Statistical Parameter** | | |
| --- | --- | --- | --- | --- | --- | --- | --- | --- | --- | --- | --- | --- | --- | --- | --- | --- | --- | --- | --- | --- | --- | --- | --- | --- | --- | --- |
|  |  |  |  | **1** | **2** | **3** | **4** | **5** | **6** | **7** | **8** | **9** | **10** | **11** | **12** | **13** | **14** | **15** | **16** | **17** | **18** | **19** | **20** | **Mean** | **Standard Deviation** | **Variable Coefficient (%)** |
| Ala | minimum | 52.452 | 274.04 | 244.20 | 245.03 | 269.63 | 261.65 | 268.44 | 262.17 | 256.98 | 255.97 | 256.98 | 255.97 | 270.96 | 266.73 | 272.98 | 263.62 | 265.32 | 240.17 | 238.29 | 254.64 | 256.06 | 238.49 | 257.21 | 10.97 | 4.27 |
|  | maximum | 1322.428 | 853.62 | 799.89 | 788.47 | 914.53 | 807.93 | 922.98 | 814.45 | 928.04 | 940.62 | 928.04 | 940.62 | 866.10 | 860.36 | 837.64 | 920.97 | 890.40 | 891.71 | 849.18 | 860.33 | 871.24 | 872.11 | 860.33 | 19.65 | 2.28 |
| Arg | minimum | 1.263 | 24.84 | 23.64 | 24.15 | 25.35 | 22.62 | 24.82 | 27.59 | 23.89 | 24.63 | 23.89 | 24.63 | 25.25 | 24.77 | 24.62 | 25.27 | 24.88 | 22.21 | 23.02 | 21.59 | 24.34 | 17.70 | 23.94 | 1.96 | 8.18 |
|  | maximum | 161.749 | 177.02 | 172.05 | 175.04 | 195.57 | 161.26 | 202.33 | 170.58 | 189.69 | 194.94 | 189.69 | 194.94 | 182.46 | 188.75 | 180.57 | 178.84 | 178.94 | 189.67 | 175.09 | 181.66 | 191.71 | 202.11 | 184.79 | 10.94 | 5.92 |
| Cit | minimum | 3.086 | 27.27 | 24.94 | 25.09 | 23.62 | 23.88 | 24.02 | 23.86 | 24.21 | 25.42 | 28.21 | 25.42 | 24.40 | 23.84 | 25.52 | 24.09 | 23.94 | 24.38 | 26.31 | 25.81 | 24.35 | 29.70 | 25.05 | 1.55 | 6.20 |
|  | maximum | 97.323 | 109.19 | 102.99 | 103.33 | 94.47 | 99.93 | 105.57 | 103.51 | 99.11 | 95.12 | 99.11 | 95.12 | 98.53 | 94.80 | 94.58 | 97.48 | 102.36 | 102.14 | 98.14 | 96.66 | 105.71 | 108.07 | 99.84 | 4.17 | 4.17 |
| Gly | minimum | 53.660 | 1372.33 | 1197.78 | 1198.52 | 1332.83 | 1323.46 | 1309.15 | 1330.41 | 1162.66 | 1158.21 | 1162.66 | 1158.21 | 1342.44 | 1336.17 | 1287.33 | 1353.69 | 1333.01 | 1215.27 | 1206.69 | 1218.14 | 1245.24 | 1223.10 | 1254.75 | 72.22 | 5.76 |
|  | maximum | 2243.583 | 2357.24 | 2106.21 | 2154.45 | 2221.18 | 2204.17 | 2463.41 | 2458.01 | 2334.94 | 2254.74 | 2334.94 | 2254.74 | 2348.82 | 2341.66 | 2287.73 | 2280.82 | 2420.56 | 2435.39 | 2325.90 | 2316.48 | 2531.96 | 2555.97 | 2331.60 | 119.24 | 5.11 |
| Leu | minimum | 36.705 | 180.57 | 160.54 | 160.46 | 175.73 | 172.92 | 176.29 | 171.04 | 166.16 | 164.04 | 166.16 | 164.04 | 178.54 | 176.76 | 176.87 | 176.29 | 177.09 | 158.03 | 157.17 | 164.64 | 168.37 | 150.02 | 168.06 | 8.21 | 4.89 |
|  | maximum | 940.453 | 476.34 | 455.14 | 455.50 | 450.07 | 514.46 | 451.86 | 512.10 | 512.25 | 501.98 | 512.25 | 501.98 | 513.15 | 507.00 | 481.08 | 479.81 | 505.53 | 502.98 | 486.54 | 484.59 | 535.71 | 537.65 | 495.08 | 26.28 | 5.31 |
| Met | minimum | 5.047 | 35.28 | 31.31 | 30.19 | 35.13 | 32.37 | 34.45 | 33.88 | 32.09 | 32.75 | 32.09 | 32.75 | 32.14 | 34.36 | 33.74 | 34.26 | 34.29 | 32.75 | 32.37 | 32.26 | 33.18 | 28.28 | 32.73 | 1.60 | 4.89 |
|  | maximum | 216.184 | 108.39 | 101.27 | 101.50 | 101.60 | 115.92 | 114.63 | 100.06 | 115.18 | 114.07 | 115.18 | 114.07 | 115.15 | 115.26 | 107.74 | 108.33 | 113.29 | 113.74 | 112.88 | 111.07 | 119.93 | 120.75 | 111.58 | 6.16 | 5.52 |
| Phe | minimum | 11.240 | 87.77 | 77.44 | 76.39 | 83.06 | 83.22 | 87.78 | 86.69 | 78.67 | 78.80 | 78.67 | 78.80 | 84.06 | 85.28 | 85.74 | 84.73 | 83.07 | 76.73 | 75.77 | 79.69 | 79.99 | 73.84 | 80.92 | 4.04 | 4.99 |
|  | maximum | 381.085 | 318.29 | 300.43 | 300.38 | 300.37 | 346.93 | 346.49 | 298.67 | 325.79 | 323.43 | 325.79 | 323.43 | 317.61 | 314.17 | 329.35 | 332.53 | 328.20 | 332.85 | 317.74 | 317.59 | 351.63 | 345.10 | 323.92 | 16.18 | 5.00 |
| Pro | minimum | 29.056 | 214.22 | 194.06 | 193.93 | 205.05 | 199.11 | 210.06 | 214.19 | 199.44 | 197.31 | 199.44 | 197.31 | 207.63 | 211.49 | 212.08 | 212.80 | 211.57 | 189.22 | 189.13 | 201.26 | 197.81 | 176.27 | 200.96 | 9.83 | 4.89 |
|  | maximum | 855.058 | 499.68 | 474.78 | 468.53 | 530.27 | 470.97 | 532.84 | 468.30 | 534.39 | 532.19 | 534.39 | 532.19 | 500.71 | 514.56 | 527.09 | 529.44 | 528.17 | 516.67 | 502.89 | 503.24 | 557.10 | 558.10 | 515.84 | 27.56 | 5.34 |
| Tyr | minimum | 14.144 | 78.84 | 73.20 | 76.66 | 77.71 | 77.43 | 76.57 | 76.08 | 74.95 | 72.58 | 72.58 | 74.95 | 72.89 | 75.82 | 72.07 | 76.63 | 78.59 | 69.89 | 75.33 | 73.00 | 71.58 | 65.92 | 74.22 | 3.04 | 4.10 |
|  | maximum | 549.908 | 338.84 | 311.82 | 313.27 | 363.28 | 311.03 | 314.22 | 368.49 | 311.83 | 315.53 | 311.83 | 315.53 | 329.65 | 324.99 | 324.12 | 316.82 | 346.56 | 339.48 | 328.54 | 323.46 | 359.98 | 361.00 | 329.57 | 19.72 | 5.98 |
| Val | minimum | 16.536 | 195.38 | 172.16 | 174.12 | 180.51 | 190.16 | 191.97 | 179.69 | 191.41 | 192.90 | 191.41 | 192.90 | 201.60 | 187.05 | 186.96 | 184.32 | 185.10 | 168.12 | 173.05 | 184.04 | 184.28 | 163.95 | 183.79 | 9.57 | 5.21 |
|  | maximum | 585.907 | 488.50 | 463.26 | 459.33 | 454.66 | 520.04 | 451.46 | 525.03 | 557.08 | 555.42 | 557.08 | 555.42 | 480.90 | 482.49 | 542.89 | 552.23 | 492.17 | 499.62 | 488.50 | 490.58 | 547.55 | 558.33 | 511.70 | 39.44 | 7.71 |
| C0 | minimum | 4.825 | 32.13 | 28.02 | 29.71 | 28.67 | 30.00 | 31.41 | 29.46 | 30.71 | 30.23 | 30.71 | 30.23 | 31.30 | 31.29 | 31.90 | 30.64 | 29.53 | 28.29 | 30.46 | 28.51 | 31.34 | 29.86 | 30.11 | 1.12 | 3.71 |
|  | maximum | 119.651 | 112.31 | 104.18 | 88.66 | 119.24 | 119.16 | 100.38 | 98.36 | 119.47 | 119.47 | 119.47 | 119.47 | 109.81 | 110.65 | 114.70 | 117.78 | 111.96 | 117.45 | 111.26 | 111.67 | 120.73 | 124.06 | 112.90 | 8.99 | 7.97 |
| C2 | minimum | 2.365 | 22.64 | 21.35 | 22.30 | 24.04 | 22.65 | 23.94 | 23.78 | 20.86 | 20.30 | 20.86 | 20.30 | 23.39 | 23.22 | 22.32 | 23.67 | 24.14 | 19.82 | 20.85 | 20.97 | 21.66 | 20.98 | 22.07 | 1.44 | 6.53 |
|  | maximum | 109.634 | 95.53 | 94.02 | 92.73 | 105.67 | 95.24 | 99.89 | 90.82 | 104.82 | 103.93 | 104.82 | 103.93 | 101.98 | 94.39 | 99.45 | 100.90 | 102.68 | 106.52 | 97.09 | 99.27 | 107.95 | 110.85 | 100.85 | 5.46 | 5.42 |
| C3 | minimum | 0.324 | 4.23 | 4.09 | 4.04 | 4.45 | 4.26 | 4.11 | 4.32 | 3.75 | 3.74 | 3.75 | 3.74 | 4.32 | 4.14 | 4.16 | 4.28 | 4.16 | 3.72 | 3.91 | 4.11 | 3.98 | 3.98 | 4.05 | 0.22 | 5.53 |
|  | maximum | 29.524 | 15.64 | 15.35 | 15.64 | 17.38 | 13.78 | 14.77 | 16.52 | 16.39 | 16.63 | 16.39 | 16.63 | 16.60 | 16.32 | 15.70 | 15.95 | 16.92 | 16.55 | 16.02 | 15.62 | 17.89 | 17.70 | 16.24 | 0.96 | 5.90 |
| C4 | minimum | 0.047 | 2.04 | 1.81 | 1.90 | 2.04 | 2.00 | 1.98 | 1.94 | 1.86 | 1.83 | 1.86 | 1.83 | 2.00 | 2.07 | 1.99 | 2.08 | 2.13 | 1.93 | 1.88 | 1.84 | 1.83 | 2.02 | 1.94 | 0.10 | 5.04 |
|  | maximum | 9.616 | 9.19 | 8.88 | 8.87 | 8.57 | 9.48 | 9.77 | 8.61 | 9.95 | 9.81 | 9.95 | 9.81 | 9.35 | 9.50 | 9.65 | 9.75 | 9.99 | 9.99 | 9.16 | 9.62 | 9.98 | 10.17 | 9.54 | 0.48 | 5.08 |
| C5 | minimum | 0.039 | 1.03 | 0.99 | 0.98 | 1.09 | 0.97 | 1.00 | 1.03 | 0.94 | 0.93 | 0.94 | 0.93 | 1.04 | 1.05 | 0.99 | 1.10 | 1.16 | 0.99 | 1.00 | 0.97 | 1.01 | 1.22 | 1.02 | 0.08 | 7.53 |
|  | maximum | 6.548 | 3.09 | 3.06 | 3.14 | 3.03 | 2.95 | 3.51 | 3.31 | 3.19 | 3.19 | 3.19 | 3.19 | 3.37 | 3.27 | 3.24 | 3.18 | 3.58 | 3.51 | 3.34 | 3.28 | 3.72 | 3.72 | 3.30 | 0.21 | 6.50 |
| C5DC | minimum | 0.018 | 0.85 | 0.84 | 0.84 | 0.88 | 0.84 | 0.80 | 0.91 | 0.77 | 0.78 | 0.77 | 0.78 | 0.77 | 0.90 | 0.82 | 0.91 | 0.92 | 0.79 | 0.78 | 0.81 | 0.78 | 0.82 | 0.83 | 0.05 | 6.34 |
|  | maximum | 6.606 | 3.00 | 2.95 | 2.93 | 3.35 | 2.84 | 3.26 | 2.77 | 3.22 | 3.29 | 3.22 | 3.29 | 3.05 | 2.98 | 3.14 | 3.28 | 3.35 | 3.39 | 3.08 | 2.95 | 3.59 | 3.50 | 3.17 | 0.22 | 7.00 |
| C6 | minimum | 0.009 | 0.74 | 0.71 | 0.67 | 0.71 | 0.76 | 0.77 | 0.71 | 0.65 | 0.62 | 0.65 | 0.62 | 0.76 | 0.74 | 0.74 | 0.75 | 0.82 | 0.67 | 0.68 | 0.69 | 0.72 | 0.82 | 0.71 | 0.06 | 8.14 |
|  | maximum | 7.006 | 3.02 | 2.91 | 2.97 | 3.27 | 3.36 | 2.92 | 2.87 | 3.17 | 3.22 | 3.17 | 3.22 | 3.30 | 3.19 | 3.07 | 3.12 | 3.37 | 3.38 | 3.16 | 3.23 | 3.60 | 3.43 | 3.20 | 0.19 | 5.86 |
| C8 | minimum | 0.009 | 0.65 | 0.60 | 0.59 | 0.62 | 0.64 | 0.67 | 0.69 | 0.61 | 0.60 | 0.61 | 0.60 | 0.65 | 0.67 | 0.63 | 0.70 | 0.68 | 0.62 | 0.60 | 0.58 | 0.66 | 0.65 | 0.63 | 0.04 | 5.66 |
|  | maximum | 6.850 | 2.81 | 2.72 | 2.79 | 3.13 | 2.78 | 3.14 | 2.68 | 3.05 | 2.99 | 3.05 | 2.99 | 2.79 | 2.89 | 3.05 | 2.82 | 3.05 | 3.00 | 3.05 | 2.93 | 3.24 | 3.34 | 2.97 | 0.17 | 5.85 |
| C10 | minimum | 0.009 | 0.81 | 0.77 | 0.75 | 0.89 | 0.84 | 0.85 | 0.83 | 0.73 | 0.74 | 0.73 | 0.74 | 0.86 | 0.84 | 0.79 | 0.83 | 0.84 | 0.80 | 0.79 | 0.78 | 0.80 | 0.84 | 0.80 | 0.05 | 5.95 |
|  | maximum | 6.753 | 2.78 | 2.73 | 2.73 | 2.64 | 3.15 | 2.77 | 3.02 | 2.99 | 3.05 | 3.19 | 3.05 | 2.95 | 2.99 | 2.98 | 3.00 | 3.14 | 3.11 | 3.04 | 3.01 | 3.19 | 3.04 | 2.99 | 0.16 | 5.23 |
| C12 | minimum | 0.009 | 1.58 | 1.49 | 1.48 | 1.63 | 1.72 | 1.67 | 1.63 | 1.43 | 1.42 | 1.43 | 1.42 | 1.65 | 1.54 | 1.55 | 1.65 | 1.64 | 1.53 | 1.54 | 1.53 | 1.51 | 1.73 | 1.56 | 0.10 | 6.39 |
|  | maximum | 7.057 | 6.00 | 5.98 | 5.99 | 6.95 | 5.96 | 5.88 | 6.95 | 6.32 | 6.27 | 6.32 | 6.27 | 6.56 | 6.44 | 6.25 | 6.42 | 6.79 | 6.65 | 6.63 | 6.55 | 7.06 | 7.22 | 6.47 | 0.38 | 5.93 |
| C14 | minimum | 0.019 | 1.45 | 1.31 | 1.32 | 1.51 | 1.54 | 1.49 | 1.56 | 1.34 | 1.29 | 1.34 | 1.29 | 1.32 | 1.46 | 1.45 | 1.51 | 1.48 | 1.40 | 1.36 | 1.39 | 1.42 | 1.62 | 1.42 | 0.10 | 6.95 |
|  | maximum | 6.775 | 5.66 | 5.54 | 5.64 | 6.57 | 5.70 | 6.52 | 5.57 | 5.96 | 5.97 | 5.96 | 5.97 | 5.98 | 5.89 | 5.92 | 6.13 | 6.23 | 6.17 | 6.19 | 6.07 | 6.61 | 6.49 | 6.05 | 0.32 | 5.26 |
| C16 | minimum | 0.194 | 4.55 | 4.26 | 4.21 | 4.88 | 4.78 | 4.77 | 4.75 | 4.09 | 4.21 | 4.09 | 4.21 | 4.64 | 4.36 | 4.28 | 4.70 | 4.63 | 4.24 | 4.22 | 4.49 | 4.59 | 4.97 | 4.47 | 0.28 | 6.30 |
|  | maximum | 17.335 | 15.63 | 15.45 | 15.34 | 15.84 | 18.50 | 18.46 | 16.02 | 16.18 | 16.41 | 16.18 | 16.41 | 16.15 | 16.02 | 16.57 | 16.46 | 16.98 | 17.04 | 17.04 | 16.88 | 17.89 | 18.29 | 16.71 | 0.94 | 5.63 |
| C18 | minimum | 0.049 | 2.27 | 2.13 | 2.14 | 2.41 | 2.36 | 2.37 | 2.42 | 2.08 | 2.07 | 2.08 | 2.07 | 2.31 | 2.19 | 2.21 | 2.31 | 2.26 | 2.02 | 2.06 | 2.21 | 2.25 | 2.34 | 2.21 | 0.13 | 5.82 |
|  | maximum | 10.424 | 4.75 | 4.62 | 4.57 | 4.71 | 5.48 | 5.49 | 4.72 | 4.68 | 4.73 | 4.68 | 4.73 | 4.78 | 4.69 | 4.75 | 4.77 | 4.99 | 4.94 | 4.79 | 4.76 | 5.22 | 5.15 | 4.86 | 0.27 | 5.52 |
